# Case Report: Pleuropulmonary Blastoma in a 2.5-Year-Old Boy: ^18^F-FDG PET/CT Findings

**DOI:** 10.3389/fnume.2021.780485

**Published:** 2021-12-14

**Authors:** Dan Ruan, Long Sun

**Affiliations:** ^1^Department of Nuclear Medicine, Zhongshan Hospital, Fudan University (Xiamen Branch), Xiamen, China; ^2^Department of Nuclear Medicine and Minnan PET Center, Xiamen Cancer Hospital, The First Affiliated Hospital of Xiamen University, Xiamen, China

**Keywords:** pleuropulmonary blastoma, ^18^F-FDG PET/CT, children with rare malignancy, conventional imaging, targeted biopsy

## Abstract

Pleuropulmonary blastoma (PPB) is a rare invasive primary malignancy in the thoracic cavity that occurs mainly in infants and children. It is often misdiagnosed and not treated correctly and promptly due to the lack of specificity of clinical symptoms and conventional imaging presentations. We report a 2.5-year-old boy who underwent X-ray chest radiography, chest CT, and ^18^F-FDG PET/CT. PET/CT images demonstrated a sizeable cystic-solid mass with heterogeneous increased glucose metabolism in the left thoracic cavity. The diagnosis of PPB (type II) was finally confirmed by a CT-guided puncture biopsy of the active tumor tissue. This case highlights the critical role of ^18^F-FDG PET/CT in the diagnosis of PPB in children.

## Introduction

Pleuropulmonary blastoma (PPB) is a rare malignant tumor of pleura or lung origin, often located in the lung periphery and invading the chest wall, mediastinum, thoracic vessels, lymph nodes, and diaphragm (1). PPB is one of the more common types of primary lung malignancies in infants and children. According to the stage of development, PPB is divided into three types, the earliest being purely cystic (type I), further progressing to mixed cystic-solid (type II), and finally solid (type III) (2).

The clinical symptoms of PPB are atypical, in which children often present with shortness of breath or respiratory distress, flushing, and fever and are often mistaken for respiratory tract infections, pneumothorax, or pneumonia (3, 4). When children present with this lung inflammation-like condition, the routine imaging examination on admission is a chest radiograph. Chest radiographs often show reduced lung translucency, often misdiagnosed as pneumonia combined with the children's symptoms. Chest CT is helpful in the diagnosis of PPB but provides limited diagnostic information. The 5-year survival rate of PPB is significantly related to the type, with 91% for type I, 71% for type II, and 53% for type III (5). Therefore, early diagnosis is crucial.

^18^F-FDG PET/CT in pediatric oncology has been shown to have more benefits than drawbacks and is a more sensitive and specific diagnostic tool for evaluating pediatric malignancies (6). The application of ^18^F-FDG PET/CT in PPB has rarely been reported. We present this case report mainly to demonstrate the diagnostic value of ^18^F-FDG PET/CT in pediatric PPB patients.

## Case Report

**Figure 1:**
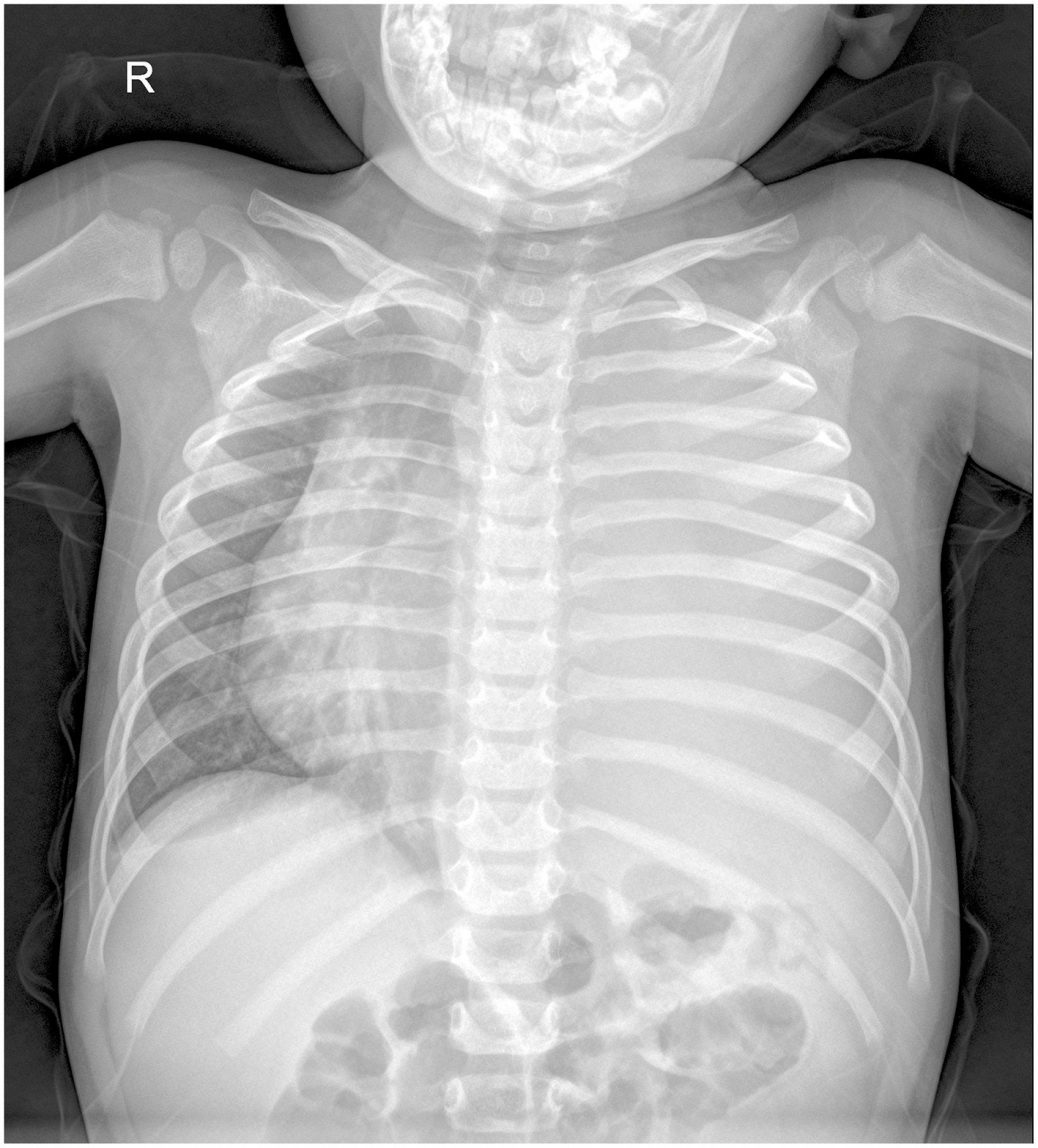
Chest X-ray showed reduced transparency of the left side of the chest with a mild shift of the mediastinum to the right.

**Figure 2:**
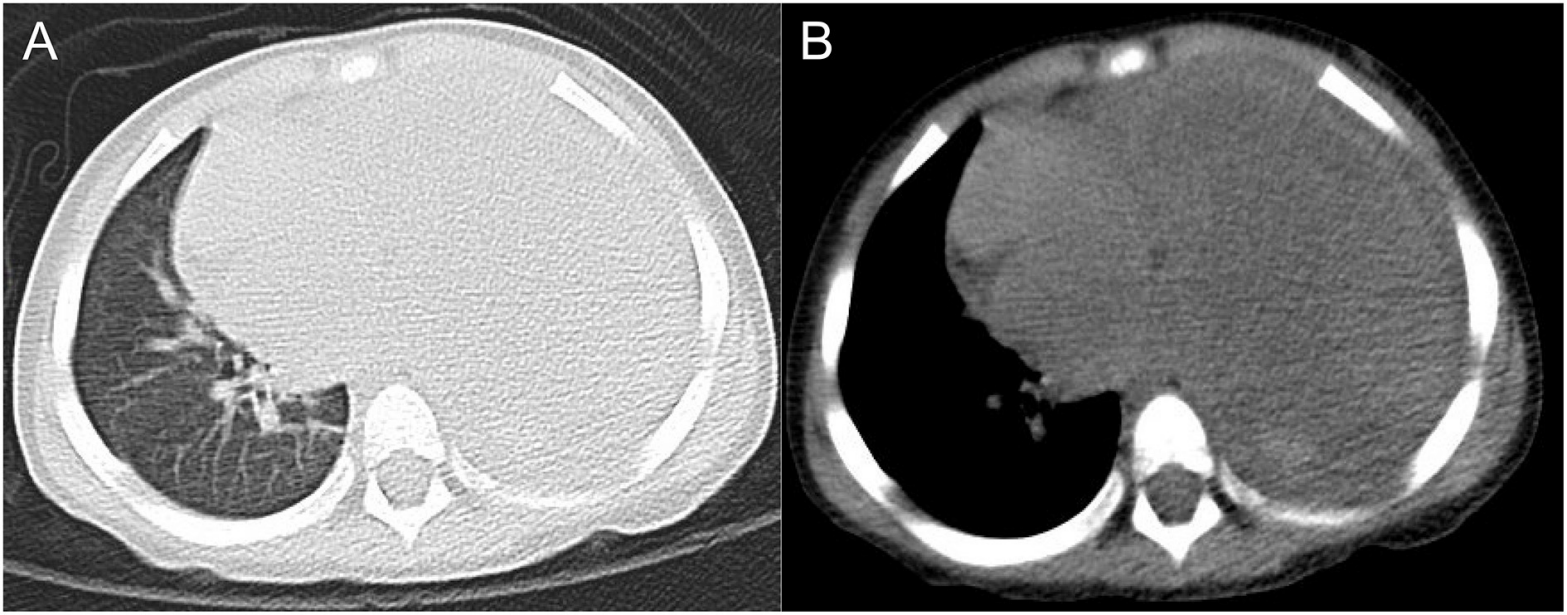
Axial lung window CT image **(A)** and mediastinal window CT image **(B)** showed a sizeable occupying lesion on the left side.

A 2.5-year-old boy presented to the hospital with an unexplained fever (maximum temperature 38.6°C). Before admission, the fever had persisted for 3 days, during which he had an occasional cough with sputum. His emergency blood tests showed a higher than normal white blood cell count (13.4 × 10^9^/L) and C-reactive protein (51 mg/L) but a normal neutrophil ratio (60.2%). He underwent a chest X-ray. A plain chest radiograph (Figure 1) showed a markedly reduced translucency throughout the left side of the chest and a mild deviation of the mediastinum to the right. The initial diagnosis was left lung inflammation with massive pleural effusion. The clinician treated him with intravenous drip cephalosporin antibiotics. However, after 2 days of treatment, there was no improvement in his condition. He was then scheduled for a CT examination of the chest. The lung window CT image (Figure 2A) showed complete solidity of the left thoracic cavity and a mild shift of the mediastinum to the right. The mediastinal window CT image (Figure 2B) revealed a large, heterogeneous density lesion in the left thoracic cavity, which consisted of multiple slightly hypodense solid components (CT value, 28–36 Hu) and multiple slightly low-density fluid components (CT value, 18–25 Hu). The lesion was poorly demarcated from the adjacent mediastinum and pleura. Subsequent blood tests, sputum tests, sputum cultures, and pleural fluid tests showed negative results for bacterial, tuberculosis, and fungal infections. However, his serum tumor marker tests showed that cancer antigen (CA) 125 (65.7 U/mL) and CA199 (218.62 U/mL) were above the normal range. He and his family did not have any history of malignancy.

**Figure 3:**
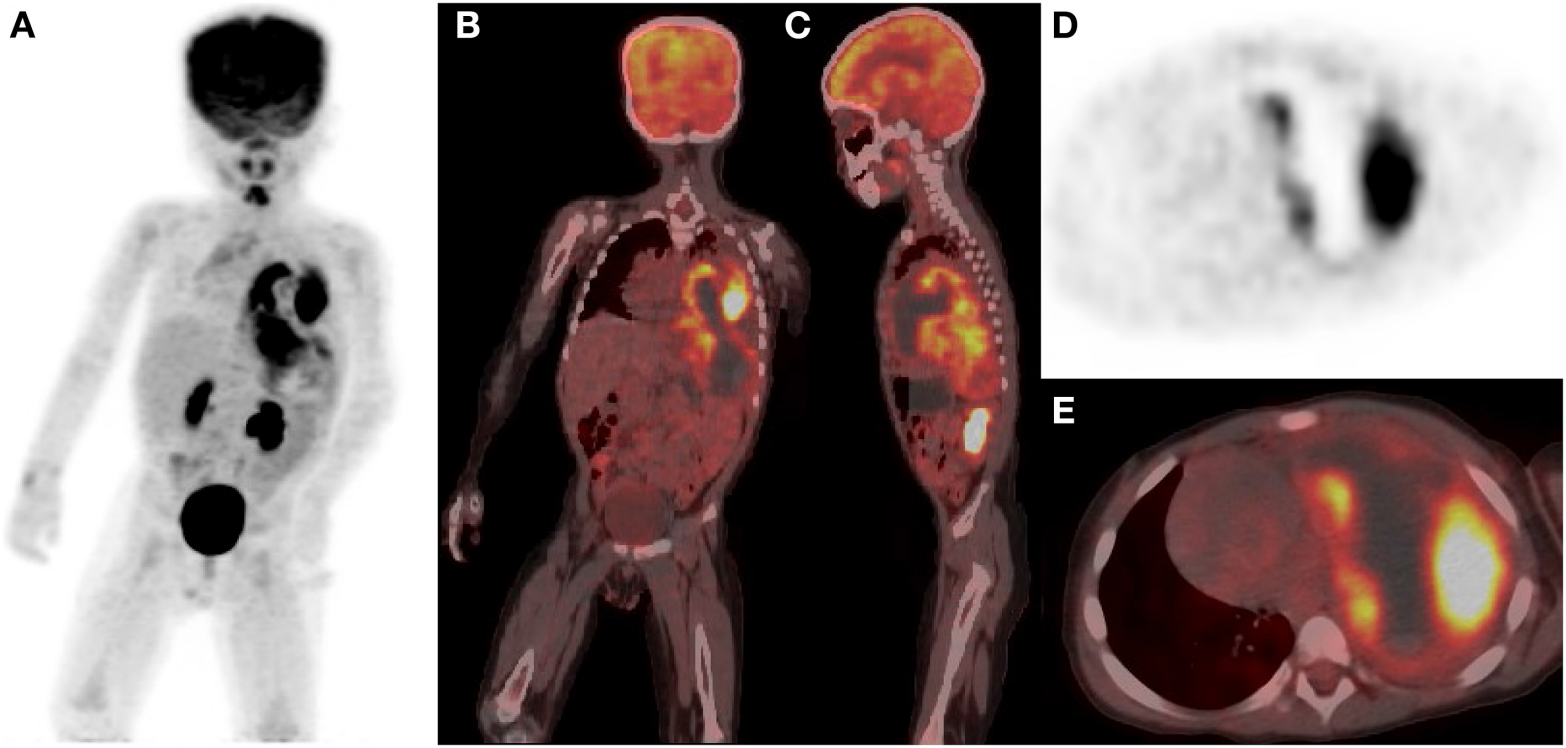
^18^F-FDG PET/CT showed a large heterogeneous density, heterogeneous ^18^F-FDG uptake mass occupying the entire left thoracic cavity. **(A)** is a whole-body MIP map, **(B)** is a coronal fused PET/CT image, **(C)** is a sagittal fused PET/CT image, **(D)** is an axial PET image, and E is an axial fused PET/CT image.

Three days later, he was scheduled for an ^18^F-FDG PET/CT examination. The maximum intensity projection (MIP) image (Figure 3A) showed a mass with heterogeneous ^18^F-FDG uptake occupying almost the entire left thoracic cavity. Coronal and sagittal PET/CT images (Figures 3B,C) showed significant ^18^F-FDG uptake in the solid portion and lack of ^18^F-FDG avidity in the cystic portion of the large mass, along with a measured longitudinal height of 14.2 cm. Axial PET image (Figure 3D) and PET/CT image (Figure 3E) showed intense ^18^F-FDG uptake in the solid component (SUVmax 7.7) and no ^18^F-FDG uptake in the cystic portion, and the maximum cross-sectional area of the mass at this axial level was 11.6 × 9.0 cm. In addition, the axial PET/CT image showed invasion of the adjacent mediastinal pleura and the pleura of the adjacent left chest wall. Whole-body PET/CT showed no signs of distant metastases.

**Figure 4:**
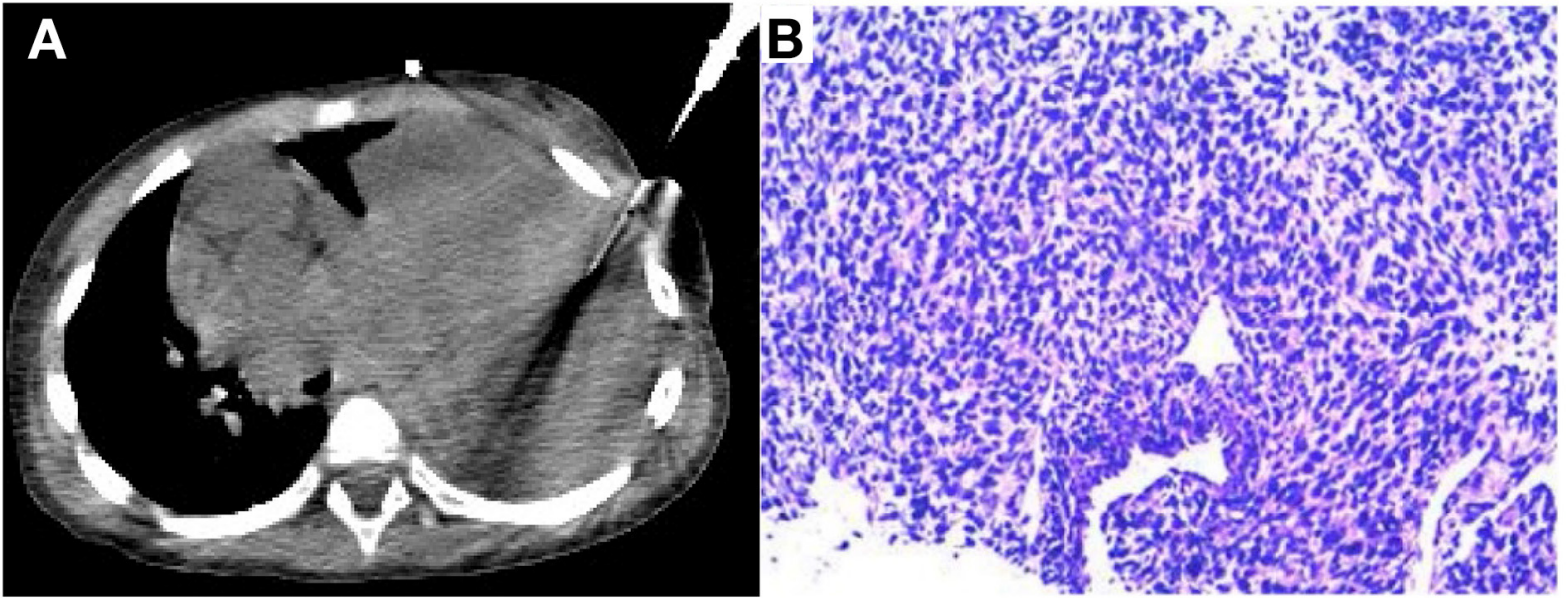
CT-guided puncture biopsy of the active solid component of the mass **(A)**. The final pathological diagnosis was pleuropulmonary blastoma (type II) with rhabdomyosarcoma differentiated mesenchyme **(B)**.

After completing the PET/CT examination, this child underwent a CT-guided puncture biopsy (Figure 4A). Based on the information provided by the ^18^F-FDG PET/CT, a solid portion of the tumor with high glucose metabolic activity was selected for puncture biopsy to obtain sufficient validated tumor tissue samples. The final pathologic diagnosis was pleuropulmonary blastoma with rhabdomyosarcoma differentiation (Figure 4B). After the diagnosis was made clear, the child was transferred to an outside hospital for further treatment.

## Discussion

PPB is considered a genetic disorder associated with germline DICER mutations. Research studies have shown that nearly 80% of children with PPB have DICER mutations (7).The early presentation of most children with PPB is characterized by fever, cough, and respiratory distress (8). Without proper diagnosis and treatment, the child's condition will continue to deteriorate until respiratory failure. Conventional X-ray examinations and CT scans lack specificity. Type I PPB presents as a cystic lesion on chest radiographs and CT and often needs to be differentiated from congenital pulmonary airway malformation (CPAM), congenital emphysema, and bronchial cysts (9–11). However, it is almost impossible to distinguish type I PPB from benign CPAM (types I and IV) by conventional imaging signs (10). The imaging presentation of type II PPB on conventional imaging is likewise not valued for differential diagnosis, i.e., it appears as a markedly decreased translucency or even complete opacity on one side of the chest on chest radiographs and as a cystic-solid mass of uneven density on CT.

Type II and III PPB are usually large and prone to distant metastases, commonly occurring in the brain, spinal cord, and bone (12, 13). ^18^F-FDG PET/CT can clearly show the adjacent tissue invasion and distant organ metastases. Another important aspect is the ability of ^18^F-FDG PET/CT to detect active tumor cell enrichment sites and accurately guide targeted biopsies, which was well-demonstrated in our case. In addition, ^18^F-FDG PET applied to type II PPB tumors can determine the active tumor components, determine the best surgical approach and the optimal surgery time, and assess the efficacy after chemotherapy, which conventional imaging tools cannot replace (3). A key element in diagnosing type I PPB is detecting the presence of the primitive rhabdomyocyte-forming layers (14). Conventional imaging-guided puncture biopsy is difficult to obtain the active tumor fraction. There is no report on ^18^F-FDG PET/CT application to type I PPB; therefore, it is still unknown whether ^18^F-FDG PET/CT can detect the active tumor component present in type I PPB.

Another aspect of interest is that our final pathological results showed that the tumor tissue of PPB was partially differentiated as rhabdomyosarcoma. A case of type III PPB reported by Geiger J also showed localized tumor tissue with rhabdomyosarcoma differentiation and presented high ^18^F-FDG uptake (3). There are no references for the performance of type I and type II PPB on ^18^F-FDG PET/CT, so we cannot be sure whether PPB tumor cells take up ^18^F-FDG or whether the high ^18^F-FDG uptake of type II PPB we report is caused by PPB cells alone, by partially differentiated rhabdomyosarcoma tissue alone, or by them together. It has been shown that rhabdomyosarcoma has a high glucose metabolism (average SUVmax of 7.2) and that the higher the degree of glucose uptake, the more aggressive the tumor is and the more likely it is to develop distant metastases (15, 16). Furthermore, higher glucose metabolism in rhabdomyosarcoma tends to predict a worse prognosis (17). Whether this suggests that the type II PPB with rhabdomyosarcoma differentiation we reported is also highly aggressive and has a worse prognosis remains to be further studied.

Finally, the application of ^18^F-FDG PET/CT in pediatric oncologic diseases has radiation safety issues. There are already many pediatric standardized PET/CT scanning protocols that effectively reduce radiation hazards while ensuring image quality, including reducing the dose of radionuclides used for imaging and using low radiation dose CT scanning techniques. In addition, adequate preparation related to sedation before imaging, and the use of restraint straps to immobilize the infant to avoid movement during imaging, are prerequisites to ensure that image quality is at a diagnostic level (18, 19).

## Conclusion

This case report suggests that children with PPB have atypical clinical symptoms, no specific hematologic tumor indicators, and a lack of specificity in X-ray and CT image performance. ^18^F-FDG PET/CT shows that PPB has heterogeneous glucose metabolism while demonstrating local tumor invasion and distant metastases.

## Data Availability Statement

The original contributions presented in the study are included in the article/Supplementary Material, further inquiries can be directed to the corresponding authors.

## Ethics Statement

Written informed consent was obtained from the individual(s), and minor(s)' legal guardian/next of kin, for the publication of any potentially identifiable images or data included in this article.

## Author Contributions

DR was responsible for writing this manuscript and editing the images. LS was responsible for the revision and submission of the article. All authors contributed to the article and approved the submitted version.

## Conflict of Interest

The authors declare that the research was conducted in the absence of any commercial or financial relationships that could be construed as a potential conflict of interest.

## Publisher's Note

All claims expressed in this article are solely those of the authors and do not necessarily represent those of their affiliated organizations, or those of the publisher, the editors and the reviewers. Any product that may be evaluated in this article, or claim that may be made by its manufacturer, is not guaranteed or endorsed by the publisher.
