# Supplementary material for: Case Report: Pleuropulmonary Blastoma in a 2.5-Year-Old Boy: 18F-FDG PET/CT Findings
Source: Front Nucl Med. 2021 Dec 14;1:780485. doi: 10.3389/fnume.2021.780485 (PMC11440937; doi:10.3389/fnume.2021.780485)
Supplement: Supplementary file 1 [file Data_Sheet_1.PDF]

CARE Checklist of information to include when writing a case report

CARE case report guidelines

(cc) BY-NC-ND

Topic	Item	Checklist item description	Reported on Line
Title	1	The diagnosis or intervention of primary focus followed by the words "case report"	Line 1 and 2
Key Words	2	2 to 5 key words that identify diagnoses or interventions in this case report, including "case report"	Line 26 and 27
Abstract (no references)	3a	Introduction: What is unique about this case and what does it add to the scientific literature?	Line 22 and 23
	3b	Main symptoms and/or important clinical findings	Line 19 to 23
	3c	The main diagnoses, therapeutic interventions, and outcomes	Line 23 and 24
	3d	Conclusion—What is the main "take-away" lesson(s) from this case?	Line 24 and 25
Introduction	4	One or two paragraphs summarizing why this case is unique (**may include references**)	Line 52 to 55
Patient Information	5a	De-identified patient specific information.	Line 57 to 65
	5b	Primary concerns and symptoms of the patient.	Line 57 to 59
	5c	Medical, family, and psycho-social history including relevant genetic information	Line 74
	5d	Relevant past interventions with outcomes	Line 63 40 64
Clinical Findings	6	Describe significant physical examination (PE) and important clinical findings.	Line 57 to 60
Timeline	7	Historical and current information from this episode of care organized as a timeline	Line 57 and 73
Diagnostic Assessment	8a	Diagnostic testing (such as PE, laboratory testing, imaging, surveys).	Line 59 and 74
	8b	Diagnostic challenges (such as access to testing, financial, or cultural)	Line 59 and 74
	8c	Diagnosis (including other diagnoses considered)	Line 88 to 89
	8d	Prognosis (such as staging in oncology) where applicable	Line 83 to 84
Therapeutic Intervention	9a	Types of therapeutic intervention (such as pharmacologic, surgical, preventive, self-care)	Line 88 and 89
	9b	Administration of therapeutic intervention (such as dosage, strength, duration)	NO
	9c	Changes in therapeutic intervention (with rationale)	NO
Follow-up and Outcomes	10a	Clinician and patient-assessed outcomes (if available)	NO
	10b	Important follow-up diagnostic and other test results	NO
	10c	Intervention adherence and tolerability (How was this assessed?)	NO
	10d	Adverse and unanticipated events	NO
Discussion	11a	A scientific discussion of the strengths AND limitations associated with this case report	Line 117 to 136
	11b	Discussion of the relevant medical literature **with references**.	Line 104 and 129
	11c	The scientific rationale for any conclusions (including assessment of possible causes)	Line 117 and 129
	11d	The primary "take-away" lessons of this case report (without references) in a one paragraph conclusion	Line 120 and 129
Patient Perspective	12	The patient should share their perspective in one to two paragraphs on the treatment(s) they received	NO
Informed Consent	13	Did the patient give informed consent? Please provide if requested	**Yes** ☑ **No** ☐
